# Developing a Hernia Mesh Tissue Integration Index Using a Porcine Model—A Pilot Study

**DOI:** 10.3389/fsurg.2020.600195

**Published:** 2020-11-26

**Authors:** Paul Patiniott, Brendan Stagg, Alex Karatassas, Guy Maddern

**Affiliations:** ^1^Department of Surgery, University of Adelaide, Adelaide, SA, Australia; ^2^The Queen Elizabeth Hospital (TQEH), Woodville South, SA, Australia; ^3^South Australia Pathology, University of Adelaide, Adelaide, SA, Australia

**Keywords:** tissue integration, porcine (pig) model, hernia repair mesh, abdominal wall reconstruction (AWR), implantable device, biological integration, hernia, hernia mesh

## Abstract

**Introduction:** With so many prosthetics available, it can be difficult for surgeons to choose the most appropriate hernia mesh. Successful hernia repair mandates an understanding of how the patient's inflammatory response influences surgical outcomes. Failure to appreciate the importance of the biological aspect of hernia repair can be very costly as emerging evidence supports that biofilm formation and reduction in effective mesh porosity gives rise to long-term mesh complications including fibrosis, chronic mesh infection, and pain. In this pilot study, we utilized a large animal (porcine) model to develop a numerical Mesh Tissue Integration (MTI) Index focused on visible tissue ingrowth, fibrosis, adhesion formation and resorption of mesh. The aim is to help surgeons adopt an evidence-based approach in selecting the most appropriate mesh according to its tissue ingrowth characteristics, matched to the patient to achieve improved surgical outcomes and optimal patient-centered care.

**Methods:** Two forty kg female Landrace pigs were recruited for this pilot study. A total of eight commonly used hernia mesh products and two controls measuring 5 × 5cm were surgically implanted in subrectus and intraperitoneal planes. The pigs were euthanised at 2 and 4 weeks, respectively. The abdominal wall was explanted, and the mesh specimens underwent macroscopic, histologic and biomechanical analysis, with engineering and pathology teams blinded to the mesh.

**Results:** Significant differences between the degrees of MTI were observed at 2 weeks and the distinctions were even more apparent at 4 weeks. One of the interesting incidental findings we observed is that mesh products placed in the subrectus plane displayed greater degrees of adhesion strength and integration than those placed intraperitoneally.

**Conclusion:** This pilot study is one of the first to propose a functional, biological standardized model for comparing hernia mesh products. The results are encouraging and demonstrate that this is a robust and transferrable model for assessing MTI in hernia mesh. The intention for this model is that it will be utilized synergistically with long term mesh/patient outcome registries and databases to inform improved matching of mesh to patient, particularly in the setting of the complex hernia repair and abdominal wall reconstruction.

## Introduction

Repairs of abdominal wall hernias are the most frequently performed operations in general surgery (1). The last 50 years has seen rapid advances in our understanding of the biological basis of hernia development, surgical technique for repair and, most significantly, the use of prosthetics (2). Although exact figures are unknown, it is estimated that more than 20 million prosthetic meshes are implanted worldwide each year (3, 4).

Several clinical studies have demonstrated the advantages of mesh implantation and therefore international guidelines recommend, independent from the surgical technique, the use of meshes in groin and ventral hernia repair (5–7). Today almost all groin hernias are treated with meshes (8) and the use of a prosthetic material for the surgical repair of abdominal wall hernia has almost universally become accepted as the current standard of practice (9, 10).

The modern-day surgeon is confronted with a plethora of different prosthetics from numerous manufacturers, and each year sees further meshes introduced to the market. With so many prosthetics available, it can be difficult for surgeons to choose the most appropriate mesh for their patients (2). Currently, there is no universal model that is used to compare mesh products. Blatnik and others advocate for standardized mesh labeling (11). Useful as this may be, we suggest that this information (i.e., size, composition, pore size, weight, biomechanical properties) in isolation is inadequate to form a basis for selecting which mesh is most optimal for use in our patients. The presumption is that this information can be extrapolated to predict tissue response to mesh and patient outcomes. However, several recent important studies support the notion that there is a fundamental gap in understanding the degree to which a mechanical mismatch between hernia repair materials and host tissue contributes to failure at the biomaterial-tissue interface (12).

A 2012 review into hernia mesh materials by Bilsel et al. recommends that in most instances surgeons should opt for a lightweight monofilament mesh, with large pores and minimal surface area (13). However, as outlined by Klinge and Klosterhalfen in 2013 there will never be one single ideal mesh for all purposes and mesh must be selected based on the specific functional requirements. Klinge and Klosterhalfen, describe the concept of “effective porosity.” Meshes are designed with a certain porosity which may significantly decrease due to axial load, mechanical mismatch with host tissue and instability of the polymer composition. Their research has shown that pore sizes of over 1,000 microns result in less host inflammatory reaction and less fibrosis with improved mesh integration and less scar plate formation resulting in a superior repair (14).

Factors influencing the early efficacy of a hernia repair include adequate closure of the defect, the size and strength, weight of mesh, and the type and security of the mesh fixation. Longer-term efficacy is dependent on tissue incorporation into the scaffold of the mesh, the degree of mesh tissue ingrowth affects the hernia recurrence rate, the resistance of mesh to chronic infection and tissue flexibility relevant to the functional outcome.

To assist surgeons in mesh selection we aim to develop a numerical mesh-tissue integration (MTI) index as originally proposed by Karatassas et al. (15). Analysis was performed using specific macroscopic, microscopic, and biological testing techniques based on several of the established guidelines from the International Organization for Standardization (ISO). The ISO sets the standards for evaluation of biomaterials, in particular part 10993-6 which specifies test methods for the assessment of the local effects after implantation of biomaterials intended for use in medical devices (16).

The aim of this proof-of-concept pilot study was to investigate the viability of this method utilizing a porcine animal model to develop a viable MTI Index. This index will function as a standardized tool to assist surgeons in selecting the most appropriate mesh according to tissue ingrowth characteristics matched to the patient—a scientific, reproducible evidence-based approach to achieving improved surgical outcomes in hernia patients.

## Methods

This was a large animal (porcine) pilot study to evaluate the safety and efficacy of commonly used mesh products for the treatment of abdominal wall hernia in patients in turn providing the scientific foundation and appropriate model for the development of a functional MTI index.

The relevant institutional ethics approval was obtained, and the study was conducted at The Large Animal Research and Imaging Facility (LARIF) of the South Australian Health and Medical Research Institute (SAHMRI) located at Gilles Plains, South Australia.

Two white female Landrace pigs weighing 37 and 40 kg, respectively, were enrolled in this study. The animals were allowed to socialize and acclimatize to the facility for 2 weeks prior to undergoing any procedures. They had access to fresh water, nutrition on a calorie-restricted diet to avoid excessive weight gain, and daily care provided by the specialist SAHMRI animal team under the supervision of a veterinarian.

**Figure 1:**
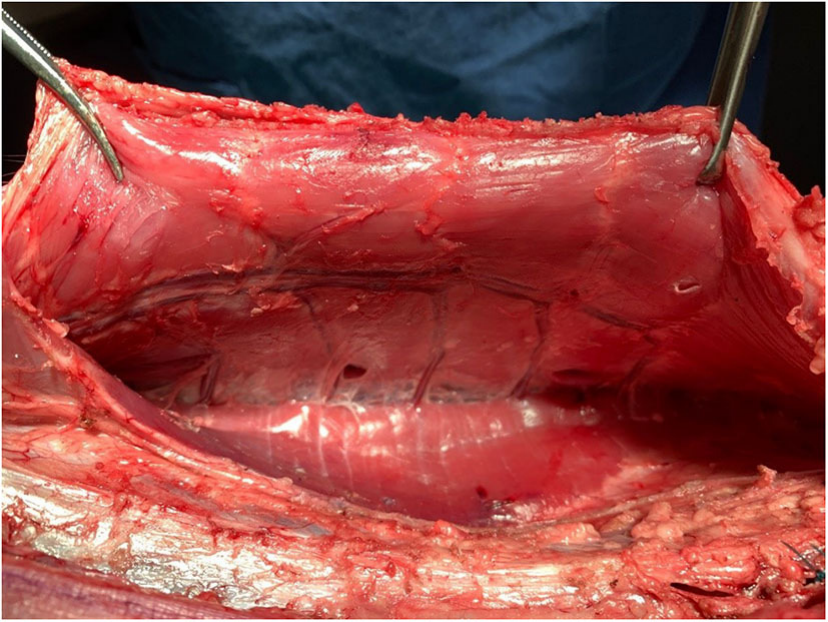
Rectorectus plane with inferior epigastric vessels and perforator bundles near linea semilunaris.

The pigs were assessed for wellness 24 h prior to surgery, then fasted from 12 h prior to surgery. Sedation was achieved with ketamine (15 mg/Kg, IM) injection and the animals subsequently anesthetized using oxygen-isoflurane inhalation. Each animal was intubated for approximately 3 h (Figure 1).

**Figure 2:**
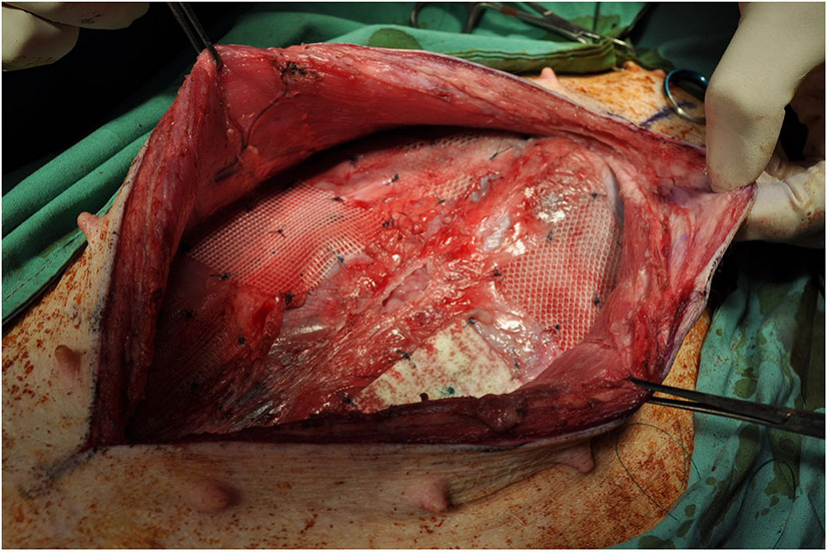
Subrectus implantation of mesh devices.

The abdomen was shaved, washed with povidone iodine and draped for surgical sterility. A 30 cm midline incision was made to gain access to the abdomen. On both sides of the abdomen a sub rectus plane was developed. Two pieces of mesh (5 × 5 cm) biosynthetic, polyester and a control were implanted in the sub rectus space on the left side of the abdomen and three pieces (5 × 5 cm) polyethylene terephthalate, polyester and polypropylene on the right side. The mesh squares were separated by 5 cm. A further six meshes (5 × 5 cm) were inserted intraperitoneally, lateral to the rectus muscle; three on the left side of the abdomen, three on the right side. All meshes were secured with 9 sutures preventing folding of mesh which may influence porosity (Figure 2).

**Figure 3:**
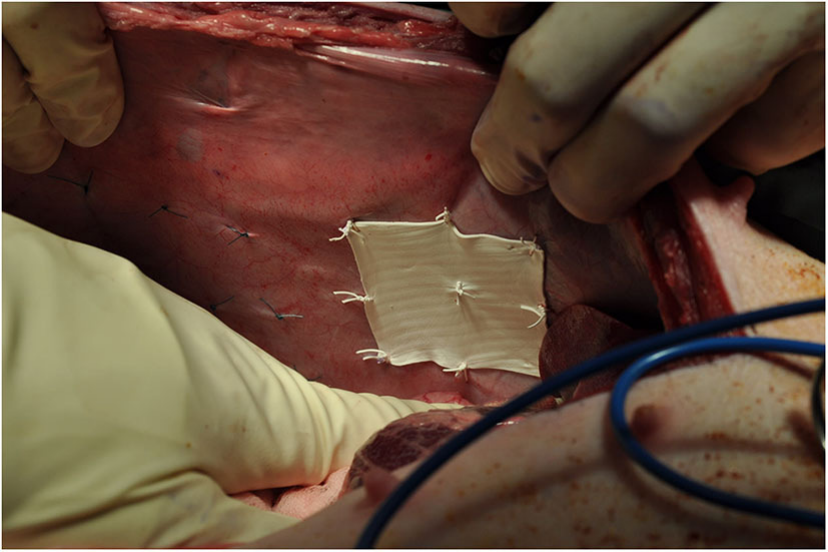
Positioning of intraperitoneal mesh (ePTFE) and control.

**Figure 4:**
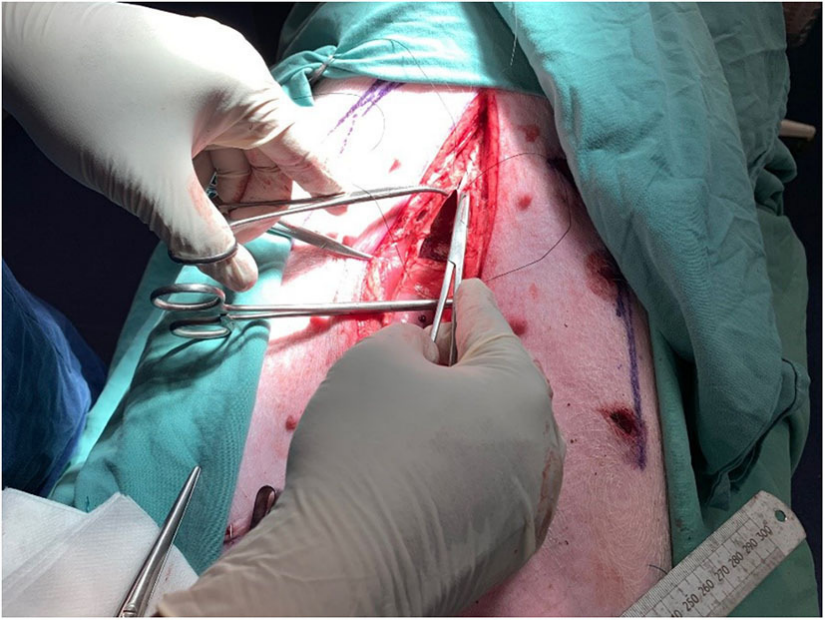
Closure of posterior layer after implantation of intraperitoneal mesh.

A total of 8 different mesh devices and 2 controls were surgically implanted in subrectus and intraperitoneal tissue planes. The controls consisted of 5 × 5 cm designated areas where 9 sutures were applied without mesh. The procedure was replicated identically for both pigs (Figures 3, 4).

**Figure 5:**
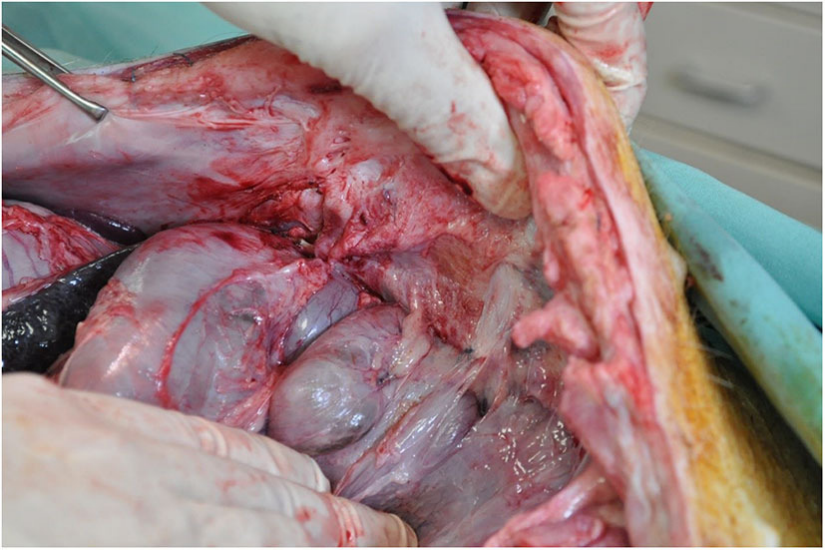
Post-mortem intraperitoneal macroscopic assessment for adhesions between mesh and viscera.

**Figure 6:**
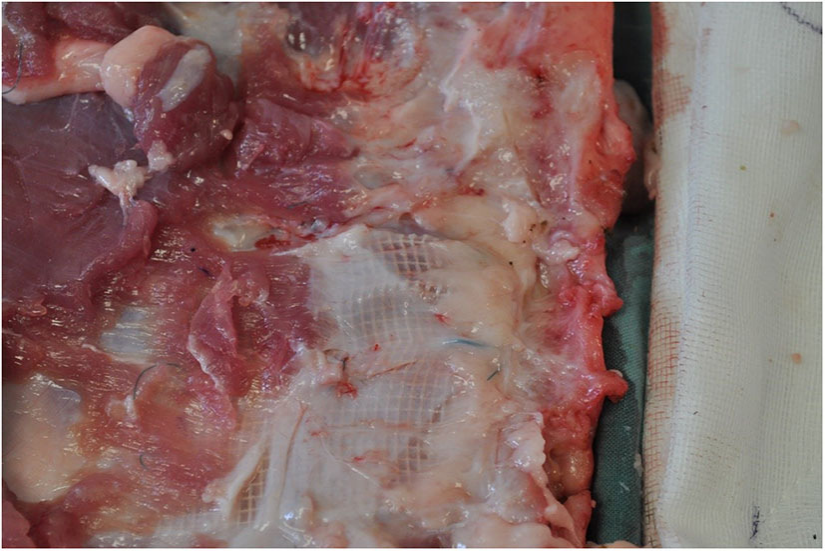
Macroscopic tissue integration of polyester mesh, subrectus plane at 2 weeks post implantation.

The pigs were euthanised utilizing 15 mLs of Lethabarb (Pentobarbitone Sodium 325 mg/mL) via intravenous cannula. The first was euthanised at 2 weeks post-implantation weighing 40 kg and second pig at 4 weeks post-implantation weighing 48.5 kg. This was to facilitate the explantation of the abdominal wall and subsequent preparation of the mesh-tissue specimens for macroscopic, histological and biomechanical analysis (Figures 5, 6).

The surgeons then performed a midline laparotomy and completed the assessment and recording of adhesion scores, the abdominal wall was separated from the animal in its entirety. Careful dissection was undertaken to define the mesh-tissue specimens which were subsequently excised from the abdominal wall and securing sutures removed. Pathology and engineering teams were blinded to the mesh being evaluated. The specimens were not labeled by brand name but instead given codes corresponding to the anatomical plane and location. For example, the mesh placed in the most anterior position on the left side of the subject in the pre-peritoneal plane was labeled PL-1, the mesh placed in the most anterior position on the right side of the subject in the intraperitoneal plane was labeled IR-1. This labeling was consistent between both pigs with the only difference being that the location of the intraperitoneal control was varied from IR-1 to IR-2 for Pig 1 and Pig 2 respectively.

The handling of biological specimens including mesh and tissue is potentially hazardous. Moreover, investigators must be wary of the inherent risk of disrupting the tissue architecture due to poor handling techniques. In order to mitigate these risks, the wearing of full sterile personal protective equipment (PPE) was enforced whenever handling specimens. In addition to this, the respective mesh-tissue specimens were carefully placed in specialized pathology containers to protect their structural integrity throughout the transportation and storage process. After the relevant macroscopic, histological, and biomechanical testing had concluded, the specimens were disposed of in the relevant medical biohazard waste disposal units in accordance with institutional guidelines which are subject to state legislation as per the Environmental Protection Act (EPA) of 1993.

The following scoring system was utilized for standardizing macroscopic assessment.

1. Visualization of degree of tissue incorporation into mesh
  1. 75–100% of mesh visible (minimal coverage of mesh with tissue)
  2. 50–75% of mesh visible
  3. 25-50% of mesh visible
  4. 0–25% mesh visible (mesh nearly or completely covered by tissue)
2. Degree of mesh shrinkage
  1. 50% shrinkage
  2. 30–50% shrinkage
  3. 10–30% shrinkage
  4. <10% shrinkage
3. Degree of adherence- force required to distract mesh from tissue
  1. Pulls away from tissue with minimal force (easily detaches with forceps)
  2. Pulls away from tissue with moderate force (detaches with use of artery forceps)
  3. Pulls away from tissue with firm force (amount of force required may partially tear mesh)
  4. Firmly attached. Cannot be pulled away
4. Adhesions to mesh—Adhesion scoring method derived from Lauder et al. (pig)—Standardized grading for adhesions to be assessed by blinded independent surgeon (17).
  Adhesion characteristics
  0 No adhesions
  1 Thin filmy adhesions
  2 More than one thin adhesion
  3 Thick adhesions with focal point
  4 Thick adhesions with planar attachment
  5 Very thick vascularised adhesions or more than one planar adhesion.

For the purpose of microscopic analysis A 50 × 10 mm strip of tissue was harvested from the center of each mesh or control site, and an orienting suture was placed at the cranial end of the specimen. After fixation in 10% neutral buffered formalin for a minimum of 12 h, the entire craniocaudal aspect of the specimen was sectioned for histological assessment. A standard 14 h processing cycle was performed, and the specimen was embedded such that the relationship between the mesh and underlying tissue layers could be examined in the plane of section. The paraffin blocks were sectioned at 4 microns, and a routine hematoxylin and eosin stain was performed.

The slides from each specimen were reviewed at scanning magnification, and formal histological assessment was performed in areas where the mesh was well-oriented and the tissue reaction was representative of the specimen as a whole. The caliber and amount of space between mesh fibers varied significantly between specimens. As such, histological assessment was performed at the mesh-tissue interface at the deep aspect of the specimen. Scoring was performed as per the guidelines of the International Organization for Standardization (ISO). ISO sets the standards for evaluation of biomaterials, specifically, ISO 10993-6 Biological evaluation of medical devices—Part 6: Tests for local effects after implantation. For each specimen, cell indices were derived as an average of 10 consecutive high power fields using a 40x objective with a field diameter of 0.52 mm (Table 1).

**Table 1:** Histological evaluation scoring system–cell type/response.

**Cell type/Response**	**0-None**	**1–Minimal**	**2–Mild**	**3–Moderate**	**4–Severe**
Polymorphonuclear cells	0	Rare,1–5/phf	5–10/phf	Heavy infiltrate	Packed
Lymphocytes	0	Rare,1–5/phf	5–10/phf	Heavy infiltrate	Packed
Plasma cells	0	Rare,1–5/phf	5–10/phf	Heavy infiltrate	Packed
Macrophages	0	Rare,1–5/phf	5–10/phf	Heavy infiltrate	Packed
Giant cells	0	Rare,1–2/phf	3–5/phf	Heavy infiltrate	Sheets
Necrosis	0	Minimal	Mild	Moderate	Severe
	**Score**				
**Response**	**0**	**1**	**2**	**3**	**4**
Neovascularisation	0	Minimal capillary proliferation, focal 1–3 buds	Group of 4–7 capillaries with supporting fibroblastic structures	Broad band of capillaries with supporting structures	Extensive band of capillaries with supporting fibroblastic structures
Fibrosis	0	Narrow band	Moderately thick band	Thick band	Extensive band
Fatty infiltrate	0	Minimal amount of fat associated with fibrosis	Several layers of fat and fibrosis	Elongated and broad accumulating of fat cells about the implant site	Extensive fat completely surrounding the implant

20 × 50 mm fresh sections were excised from the respective explanted mesh-tissue specimens and taken to The University of Adelaide, North Terrace campus for the purpose of biomechanical testing. The University Department of Engineering designed and constructed a custom-made pin loaded clamp for use with the Instron Model 1011 testing machinery, securing the tissue in order to determine the precise force (Newtons) vs. displacement (mm) curve associated with the distraction of mesh from tissue for each of the extraperitoneal and intraperitoneal mesh products in Pig 1 and Pig 2.

## Results

Macroscopically, there was no difference in visual MTI scores at 2 weeks, there was minor shrinkage of the polyethylene mesh and adherence scores were generally higher in the subrectus plane with the exception of the biosynthetic mesh. The manufacturer of the biosynthetic mesh recommends pre-peritoneal placement of this product, intraperitoneal placement was not recommended (Table 2).

**Table 2:** Macroscopic assessment–Pig 1 (2 weeks).

**Mesh**	**MTI**	**Fibrosis/Shrinkage**	**Adherence**	**Adhesions (Intraperitoneal)**
PR-1 (control)	Control	Control	Control	–
PR-2 biosynthetic	1	4	3	–
PR-3 polyester	1	4	3	–
PL-1 polyester & polylactic acid (PLA)	1	3	4	–
PL-2 polyester	1	4	3	–
PL-3 polypropylene	1	4	3	–
IR-1 ePTFE	1	4	1	0
IR-2 (control)	Control	Control	Control	0
IL-1 biosynthetic	1	4	4	2
IL-2 polyester	1	4	1	0

At 4 weeks, macroscopic MTI scores had improved for all the devices with favorable adherence, minimal to zero shrinkage, and fibrosis with the exception of PR-3 polyester, which scored comparatively lower in the 3 key domains (Table 3).

**Table 3:** Macroscopic assessment–Pig 2 (4 weeks).

**Mesh**	**MTI**	**Fibrosis/Shrinkage**	**Adherence**	**Adhesions (Intraperitoneal)**
PR-1 (control)	Control	Control	Control	–
PR-2 biosynthetic	2	4	3	–
PR-3 polyester	1	4	2	–
PL-1 polyester & polylactic acid (PLA)	3	3	4	–
PL-2 polyester	2	4	3	–
PL-3 polypropylene	3	4	3	–
IR-1 (control)	Control	Control	Control	1
IR-2 ePTFE	1	4	1	1
IL-1 biosynthetic	3	4	4	3
IL-2 polyester	1	4	1	1

**Figure 7:**
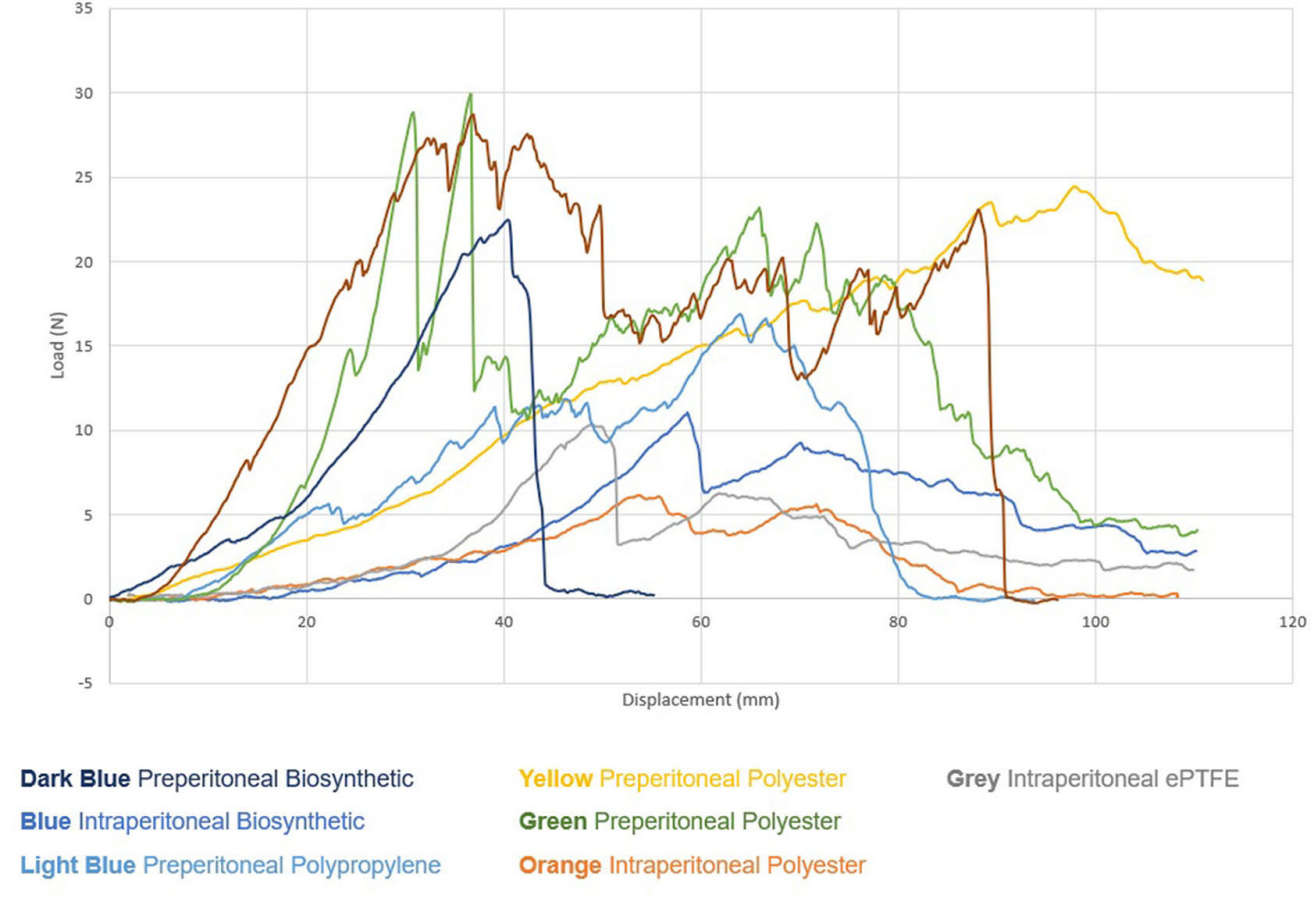
Biomechanical Testing—Load vs. Displacement Graph Pig 1 (2 weeks).

There was no substantial difference in adhesive strength between mesh-tissue specimens observed at 2 weeks (Figure 7).

**Figure 8:**
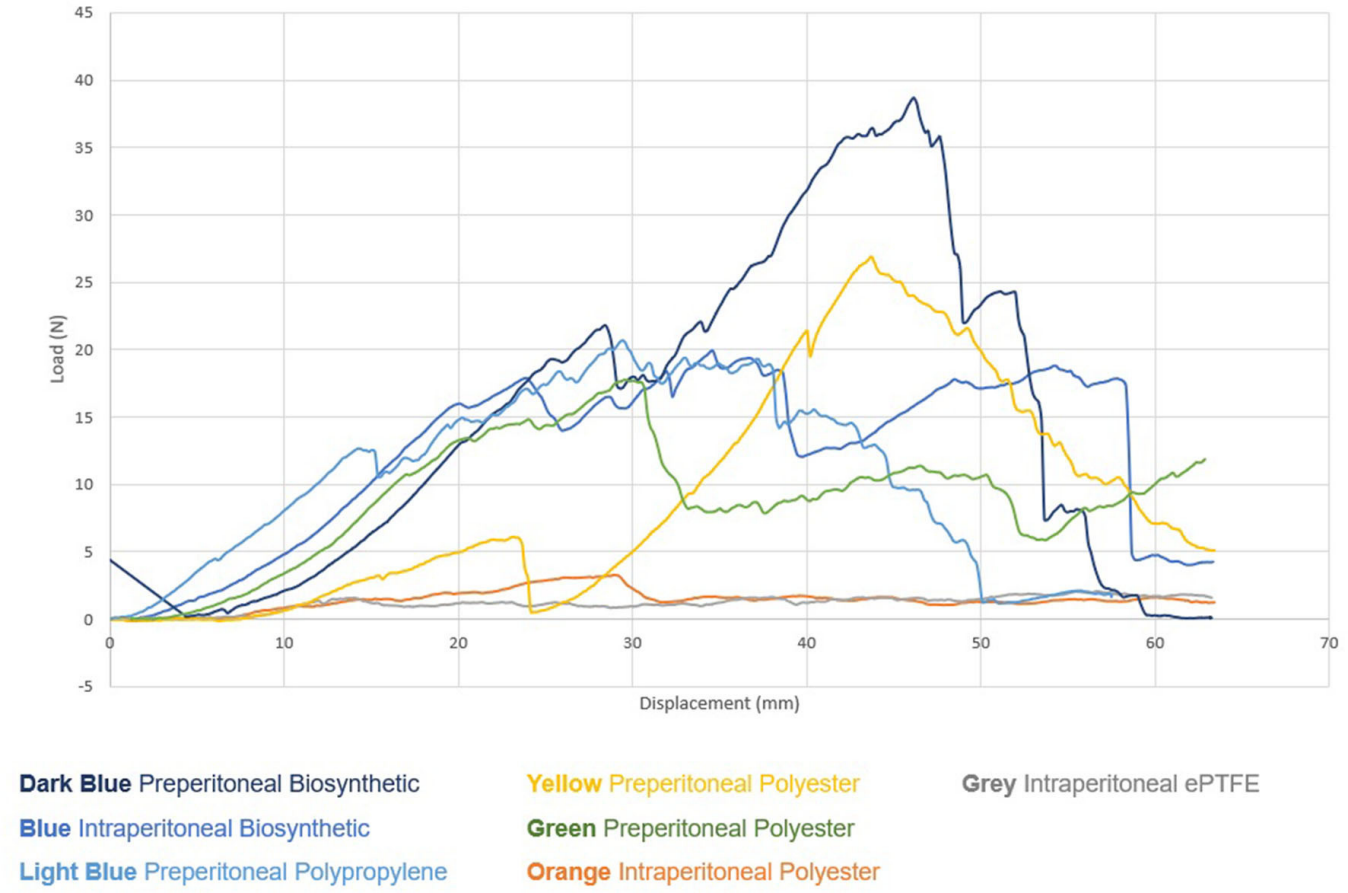
Biomechanical Testing—Load vs. Graph Pig 2 (4 weeks).

However, at 4 weeks clear trends began to emerge. Significantly, mesh devices implanted in the pre-peritoneal plane required much higher average and peak loads (Newtons) to distract the mesh from tissue when compared to the products implanted in the intraperitoneal plane (Figure 8, Table 4).

**Table 4:** Histology results–pig 1 (2 weeks).

**Pig 1 (2 weeks)**	**PR-1 *control***	**PR-2 biosynthetic**	**PR-3 polyester**	**PL-1 polyester + PLA**	**PL-2 polyester**	**PL-3 polypropylene**	**IR-1 ePTFE**	**IR-2 *control***	**IL-1 biosynthetic**	**IL-2 polyester**
**Inflammatory**										
Neutrophils	0	1	2	1	1	1	1	0	0	1
Lymphocytes	3	3	3	3	3	3	2	1	2	3
Plasma cell	0	1	1	1	1	0	0	0	0	1
Macrophages	3	3	4	3	3	3	3	1	3	3
Giant cells	0	3	3	1	2	1	3	0	3	1
Necrosis	0	1	0	1	0	1	0	0	0	0
Subtotal	6	12	13	10	10	9	9	2	8	9
**Other changes**										
Fibrosis	1	1	1	1	1	1	1	1	1	2
Fat infiltration	0	0	0	0	0	0	0	0	0	0
Neovascularisation	1	1	1	1	1	2	1	0	1	2
Subtotal	2	2	2	2	2	3	2	1	2	4
Total	8	14	15	12	12	12	11	3	10	13

Histological changes were apparent in all devices at 2 weeks and even more pronounced at 4 weeks. The biosynthetic and polypropylene devices displayed the highest histological scores at 4 weeks followed by polyester. Lower histological scores were associated wth the intraperitoneal ePTFE and composite (barrier) mesh devices (Table 5).

**Table 5:** Histology results–Pig 2 (4 weeks).

	**PR-1 *control***	**PR-2 biosynthetic**	**PR-3 polyester**	**PL-1 polyester + PLA**	**PL-2 polyester**	**PL-3 polypropylene**	**IR-1 *control***	**IR-2 ePTFE**	**IL-1 biosynthetic**	**IL-2 polyester**
**Inflammatory**										
Neutrophils	0	0	0	0	0	2	0	0	1	1
Lymphocytes	0	3	3	3	3	3	2	3	3	3
Plasma cell	0	1	0	0	0	0	0	0	0	0
Macrophages	0	3	3	3	3	3	0	3	3	3
Giant cells	0	3	3	1	1	2	0	1	3	1
Necrosis	0	1	0	0	0	1	0	0	0	0
Subtotal	0	11	9	7	7	11	2	6	10	8
**Other changes**										
Fibrosis	0	1	1	3	1	1	4	1	1	2
Fat infiltration	0	1	0	1	1	0	0	0	0	0
Neovascularisation	0	1	3	2	3	2	1	1	2	2
Subtotal	0	3	4	6	5	3	5	2	3	4
Total	0	14	13	13	12	14	7	8	13	12

## Discussion

Significant differences between the degrees of MTI were observed at 2 weeks and the distinctions were even more apparent at 4 weeks. The experimental protocol was kept as identical as possible for both timeframes, this was an intentional feature of the study design. The rationale for doing this was to minimize the number of confounding variables when investigating the changes to MTI scores over time whilst still allowing for direct comparison of the various biomaterials. One of the interesting incidental findings observed in this study is that mesh products placed in the subrectus plane displayed greater degrees of adhesion strength and integration than those placed intraperitoneally.

**Figure 9:**
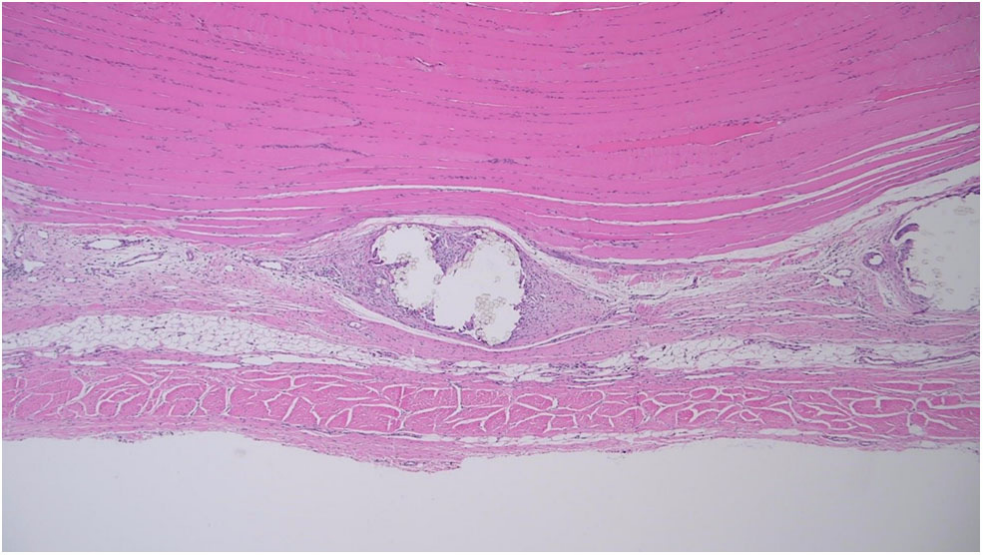
Specimen PR-2 viewed at 40 × magnification (Pig 2).

Microscopic assessment was limited by substantial variation in mesh microarchitecture between products. For example, IR-1 appeared to contain a continuous band of mesh with no perceptible gaps, whereas PL-2 contained 1 mm spaces between mesh fibers. The amount of space between mesh fibers appeared to limit the amount of room for tissue infiltration (Figure 9).

**Figure 10:**
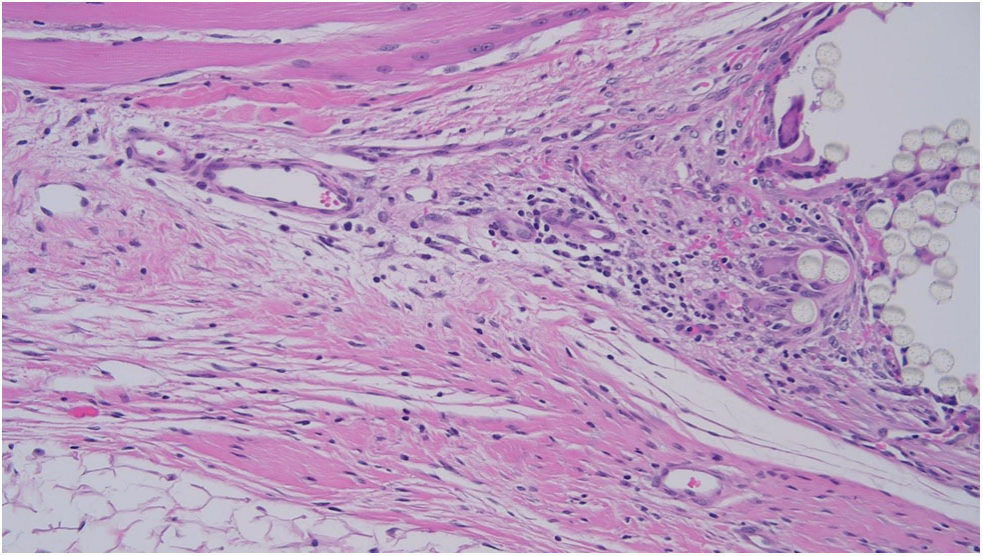
Specimen PR-2 viewed at 200 × magnification (Pig 2).

As such, the tissue response was assessed at the deep surface of the mesh to promote consistent comparison between specimens. However, for parameters such as neovascularisation and adipose tissue formation, it was more practical to examine the tissue between mesh fibers. Efforts to standardize microscopic assessment between specimens were limited by variation in mesh microarchitecture (Figure 10).

Macroscopic analysis of the subrectus mesh demonstrated that the MTI scores were on average higher in Pig 2 (4 weeks) in comparison with Pig 1 (2 weeks), whereas the lower MTI scores for the intraperitoneal mesh remained largely unchanged between the 2 pigs, with respect to both fibrosis and adherence. If additional pigs were to be recruited in future studies and endpoints were extended to 8 and 12 weeks or even longer, more datapoints would be generated and we may expect to see more significant differences in both fibrosis and adherence scores for these intraperitoneal meshes. The lack of improvement in MTI scores for the intraperitoneal mesh products between Pig 1 and 2 is not unexpected as according to the theory of effective porosity, the barrier protection significantly reduces effective porosity and hence rate and degree of MTI. No significant adhesions were noted in the intraperitoneal meshes in both pigs but this may change with longer term studies. In future studies the Jenkins scale (18), a standardized and validated adhesion scoring system could also be considered as a viable alternative to the Lauder scoring system which was utilized in this study.

Biomechanical testing and analysis of the explanted mesh-tissue specimens showed good correlation with the macroscopic index scores, however in this study there was no clear correlation with the histological findings.

Key strengths of this study included that the porcine model has proven to be very sound for the purposes of challenging our hypothesis and developing a MTI index. The study design facilitated multiple mesh products and a control to be tested simultaneously without any discernible detrimental effects to the animals. The ability to correlate results between macroscopic, microscopic and biomechanical analyses was desirable, with good concordance between macroscopic and biomechanical domains. As collaboration between investigators from several disciplines was required, blinding was essential to reducing the risk of investigator bias.

Some of the weaknesses of this study included small numbers, with only two pigs having been used in this pilot study, larger numbers in future studies will reduce the risk of bias and increase the validity. Although 4 weeks was adequate for the purposes of demonstrating model viability, ideally observation over a longer timeframe with several later study endpoints i.e. to 3, 6, or 12 months will likely result in the observation of greater variability between index scores across several domains and the emergence of stronger trends and relationships between variables. In retrospect, it was concluded that the study was made significantly more technically difficult as a result of mesh products being implanted in two anatomical planes in the same animal. Ensuring that overlapping of mesh devices did not occur was unnecessarily challenging. In future studies, appropriate consideration should be given to modifying the study to only involve implantation of mesh in one anatomical plane per animal. Future studies may also benefit from including an investigation into the effect of adjuncts on MTI; in 2007 Fortelny et al. described how a cyanoacrylate tissue sealant reduced the effective mesh porosity thereby having a detrimental effect on (MTI) (19). Conversely, in 2008 Petter-Puchner et al. utilized a rodent model to examine a fibrin-based tissue sealant which displayed a favorable MTI and adhesion profile, although more data and a longer observational period would have strengthened the study (20).

Recently, there has been increasing scrutiny in the media of hernia mesh products on an international level, public pressure on governments has resulted in the relevant regulatory bodies upgrading the classification of hernia mesh products mandating a higher degree of regulation in line with other medical prosthetic devices such as orthopedic joints and cardiac implantable devices. In view of recent events highlighting the risks associated with the use of surgical mesh devices The Australian Government Therapeutic Goods Administration (TGA) has recognized this deficiency in monitoring and regulation and recently strengthened their assessment of surgical mesh medical devices by approving regulatory amendments that reclassified all these medical devices from Class IIb (medium) to Class III (high risk) (21).

There are several successful established registries of surgical outcomes in other disciplines, including the Australian Orthopedic Association National Joint Replacement Registry (AOANJRR) (22) and the Australian Breast Device Registry (ABDR) (23) amongst others. It is lamentable that such a registry has not yet been established for hernia mesh prostheses in the field of hernia surgery.

The importance of establishing a longitudinal patient database and hernia mesh registry cannot be overstated, it will enable the identification of specific patient conditions and surgical factors which influence the tissue integration process and clinical outcome. It will also allow for the evaluation of adjuncts, such as tissue ingrowth promoters and therapies, such as those targeting the formation of biofilms. Further it will serve as a guide to surgeons when selecting the most appropriate mesh product for their hernia patient, facilitating optimal patient-centered care.

The development of a hernia MTI index as proposed in this pilot study used in conjunction with hernia mesh registries are likely to become increasingly important references for mesh manufacturers when providing preregistration data to the relevant regulatory authorities prior to market release and subsequent clinical utilization.

Longer term studies will facilitate the development of a degradation index to supplement the integration, fibrosis and adhesion indexes. It is envisaged that the MTI Index will be a useful tool for individualizing hernia treatment for patients, the ultimate intention for this model is that it will be utilized synergistically with long term mesh-patient outcome registries and databases to inform improved matching of mesh to patient, particularly in the setting of the complex hernia repair and abdominal wall reconstruction.

It is important to emphasize that this is a pilot study providing the framework for a proof-of-concept MTI index study which will involve increasing the number of subjects and observing integration over a longer timeframe. We propose proceeding to involving 10 pigs over 3 months, a larger number of data points will be essential if this is to be considered a significant model.

## Conclusion

This pilot study is one of the first to propose a functional, biological standardized model for comparing hernia mesh products. The results are encouraging and demonstrate that this is potentially a robust and transferrable model for assessing MTI in hernia mesh. A proof-of-concept study involving larger numbers and longer study endpoints is required to further improve the validity of this model.

## Data Availability Statement

The raw data supporting the conclusions of this article will be made available by the authors, without undue reservation.

## Ethics Statement

The animal study was reviewed and approved by South Australian Health & Medical Research Institute (SAHMRI) Project No. SAM323.

## Author Contributions

PP: contributed to the majority of study design, implementation, and manuscript writing. BS: responsible for the histological analysis and production of histology results and tables, reviewed paper, and contributed to the discussion section. AK: research supervisor to PP, responsible for ethics approval, study design, study implementation, and manuscript review/editing. GM: primary research supervisor to PP, responsible for research governance, oversight, and manuscript review/editing. All authors contributed to the article and approved the submitted version.

## Conflict of Interest

The authors declare that the research was conducted in the absence of any commercial or financial relationships that could be construed as a potential conflict of interest.
